# Household contact antigen-speci**fi**c TNF and IL-2 T-cell responses and impact of index case Mycobacterium tuberculosis aerosolization and HIV Co-infection

**DOI:** 10.21203/rs.3.rs-4815117/v1

**Published:** 2024-11-20

**Authors:** Lilian N. Njagi, Videlis Nduba, Wilfred Bundi Murithi, Zipporah Mwongera, Kennadi Cook, Jerphason Mecha, Robi Chacha, Kevin P. Fennelly, David J. Horne, Thomas R. Hawn

**Keywords:** Mycobacterium tuberculosis, immune response, IFN-γ-independent cytokine responses, Mtb aerosolization, cough aerosol culture

## Abstract

Exposure to pulmonary tuberculosis (PTB) culminates in heterogeneous outcomes, including variation in *Mtb* antigen-specific interferon-gamma (IFN-γ) T-cell responses. IFN-γ-independent cytokines, including tumor necrosis factor (TNF) and interleukin (IL-2), offer potential diagnostic improvements and insights into pathogenesis. We hypothesized that ESAT6/CFP10 TNF and IL-2 responses improve *Mtb* infection detection among exposed household contacts (HHCs) and are associated with index case *Mtb* aerosolization (i.e., cough aerosol culture positive for Mtb growth, CAC+]) and HIV co-infection. We enrolled individuals with PTB and their HHCs in a longitudinal study in Nairobi, Kenya. We measured TNF and IL-2 in HHCs from QuantiFERON-TB Plus TB1 tube supernatants. An additional 9.2% (25) HHCs beyond the 58.6% (129) with an IFN-γ response demonstrated an antigen-specific increase in IL-2 and TNF. HHCs of CAC + participants were more likely to have positive IL-2 (84.6% vs. 53.8%, *p* = 0.02) and IFN-γ (88.0% vs. 54.9%, *p* = 0.01), but not TNF responses, compared to CAC-negative individuals. While HIV co-infection in the index was negatively associated with IFN-γ responses in HHCs (35.7% vs. 62.3%, *p* = 0.03), IL-2 and TNF responses did not differ. Antigen-specific ESAT6/CFP10 IL-2 and TNF may increase rates of *Mtb* infection detection and provide insights into *Mtb* transmission and pathogenesis.

## Introduction

*Mycobacterium tuberculosis* (Mtb) is the leading infectious disease cause of death. [1] Most tuberculosis (TB) cases are preceded by asymptomatic *Mtb* infection. Interferon-gamma (IFN-γ), a critical pro-inflammatory cytokine and the endpoint of the interferon-gamma release assays (IGRAs) is pivotal in *Mtb* infection ascertainment. [2] In individuals heavily exposed to pulmonary TB, there is significant variation in *Mtb* infection rates, with some remaining IGRA-negative despite heavy exposure. [3–7] Alternative correlates of immunity in these individuals have been proposed. [3, 7–12] Understanding these alternative correlates of immunity may aid in developing new tools for preventing, diagnosing, and treating *Mtb*.

Interleukin (IL)-2 and tumor necrosis factor (TNF) are T helper (Th) 1 pro-inflammatory cytokines promoting cell-mediated immunity, demonstrated to have a protective and immunodiagnostic role in TB. [13–20] Both cytokines have been detected in “resisters” [10, 11, 21] and show promise in improving sensitivity in diagnosing *Mtb* infection. [22] Although these data suggest that *Mtb*-specific TNF and IL-2 responses are alternative endpoints, further research is needed to support their utility in enhancing the detection of *Mtb* infection. Additionally, differences in IFN-γ, TNF, and IL-2 responses following exposure to *Mtb* are not fully understood.

While previous studies have been conducted on cohorts with strong epidemiologic evidence of exposure to *Mtb*, [10, 11] the effect of differential levels of exposure on immunologic responses has not been explored. Differential exposure to *Mtb* may activate a distinct T-helper pathway with varying immunologic responses. [23] As *Mtb* is transmitted through aerosolization, bioaerosol sampling to estimate differential exposure may provide further insights. [23] The cough aerosol sampling system (CASS) is a cough bioaerosol sampler validated to estimate *Mtb* infectiousness. [24–26] Cough-generated aerosol cultures (CAC) are shown to predict *Mtb* infection measured by IFN-γ better than other measures of infectiousness, including sputum smear microscopy or culture. [26–28] The magnitude and directionality of the effect of the aerosolized *Mtb* on antigen-specific IFN-γ-independent cytokine responses following exposure to *Mtb* is unknown.

Further, although persons living with HIV (PLWH) are more likely to develop TB, [1] the likelihood of *Mtb* transmission to contacts is thought to be lower than that of persons without HIV. [29–31] One reason is the lower bacillary load associated with the altered pathogenesis. [30, 31] However, results on infectiousness of PLWH with TB have varied. [30, 32] In addition, previous studies evaluating the infectiousness of PLWH with TB have used conventional tests assessing sensitization to *Mtb* antigens, including the tuberculin skin test and IGRAs, to indicate *Mtb* transmission to contacts. [29–32] It has not been determined before, to our knowledge, how *Mtb* antigen-specific non-IFN-γ cytokine responses in contacts vary with HIV coinfection in the index. Non-IFN-γ responses, including IL-2 and TNF, might provide additional information on transmission dynamics beyond IFN-γ.

To address these gaps in knowledge, we determined *Mtb*-specific TNF and IL-2 responses among household contacts (HHCs) of TB participants to assess the utility in improving Mtb infection detection and the effect of aerosolized *Mtb* and HIV on the cytokine responses.

## Methods

### Study setting, design, and population.

This study used enrolment data from participants in an ongoing longitudinal cohort study designed to assess TB aerobiology, immunology, and transmission (TBAIT) in Nairobi, Kenya. [33] Adults newly diagnosed with pulmonary TB by GeneXpert and liquid culture for *Mtb* were enrolled from outpatient clinics and through a door-to-door prevalence survey (index TB participants). HHCs of any age were enrolled if they resided in the same household for at least 60 days at the time of the index patient TB diagnosis.

### Ethical approvals

The ongoing study was conducted per the principles of the Declaration of Helsinki. It was approved by the Kenya Medical Research Institute Scientific and Ethics Review Unit (048/3988) and the University of Washington Institutional Review Board (STUDY00009209). All participants provided written informed consent. All data were obtained and stored in compliance with the associated Institutional Ethics Review Unit regulations.

### Study interventions and clinical protocol.

Members of the study team administered a questionnaire to obtain clinical and demographic information, including age, sex, HIV status, alcohol use, smoking status, information on HHC sharing a bedroom with index, and anthropometric measures, including mid-upper arm circumference (MUAC), blood pressure (BP), weight, and height; Body mass index (BMI) was calculated from weight and height measurements.

Among index TB participants, sputum was collected for acid-fast bacilli (AFB) smear, culture, and GeneXpert testing. HIV testing was performed using an opt-out approach, and CD4 lymphocyte count testing was performed where applicable. Chest X-rays (CXR) were obtained, and cavitation was documented. Phlebotomy was done to measure white cell count (WBC) and C-reactive protein (CRP). Infectious aerosols were collected using a cough aerosol sampling system (CASS), as we described previously. [33] Briefly, CASS consists of a six-stage Andersen Cascade Impactor (Thermo Fischer Scientific, IL) within a larger stainless-steel chamber attached to the mouthpiece tubing from the participant and with a vacuum pump, creating an airflow through the system. [24, 25, 33] Each of the six stages holds a Middlebrook plate with 7H10 or 7H11 solid agar media on which aerosolized particles settle based on size, with the smallest particles settling on the lowest plate. [24, 25, 33] Participants coughed into the mouthpiece for 5 minutes, after which the plates were incubated at 37 °C and observed weekly for growth for a maximum of 8 weeks. [33]

HHCs underwent symptom screening (ongoing cough, hemoptysis, weight loss, chest pain, fevers, chills, or night sweats), and CXR was obtained to rule out active TB. HHCs with symptoms suggestive of TB and/or abnormal CXR underwent further TB evaluations. HHCs with microbiological confirmation of TB were enrolled as TB cases and referred for treatment. Phlebotomy for HIV testing (using an opt-out approach) and QuantiFERON-TB Plus (QFT-Plus, IGRA) testing were performed.

### Laboratory assays

#### Index TB participants:

GeneXpert MTB/RIF (Xpert MTB/RIF) or GeneXpert Ultra (Xpert Ultra) (Cepheid, Sunnyvale, CA) were performed on raw sputum samples. Results were recorded as a cycle threshold (Ct) value and semi-quantitative grade, including “Trace” for Xpert Ultra. AFB smears and culture were performed on concentrated sputum. AFB smears were examined using fluorescence microscopy (FM), and the WHO AFB scale was used for grading. Sputum cultures were performed in liquid (MGIT Becton-Dickinson, Franklin Lakes, NJ, supplemented with BD BBLTM MGITTM PANTA) using an automated BACTEC MGIT 960 machine for incubation. Positive cultures were confirmed using Ziehl-Neelsen smear microscopy, and the MGIT TBc Identification Test (Becton-Dickinson Diagnostic Instrument Systems, Sparks, MD) was used to detect MTBc-specific MPT64-antigen. Time to detect *Mtb* growth *(TTD of Mtb)* was recorded. For CAC, agar plates with infectious aerosols were incubated at 37°C for 8 weeks and observed weekly for growth. Colony-forming units (CFU) were counted where growth was identified as *Mtb*. Otherwise, agar plates were discarded as contaminated. The time to detect *Mtb* and the plates where *Mtb* was identified were documented.

#### Household contacts:

The QFT-Plus test was performed at KEMRI/CRDR following the manufacturer’s instructions (Qiagen). [34] Briefly, whole blood was collected into lithium heparin vacutainers, and 1 mL was transferred into each of the four QFT-Plus blood collection tubes within 4 hours of collection. The tubes were incubated at 37°C for 16 hours and centrifuged for 15 min at 3000 relative centrifugal force (RCF), after which plasma was collected and assessed using the standard ELISA. Results were recorded using standard QFT-Plus interpretations from the manufacturer. The remaining supernatants from the nil, mitogen, and TB1 tubes with ESAT6/CFP10 antigens were frozen at −80°c. Frozen QFT-Plus supernatants were shipped to Seattle, WA, and tested for TNF and IL-2 using sandwich ELISA according to the manufacturer’s instructions (R&D Systems Inc. Minneapolis, USA.) Briefly, the supernatants were transferred from the vials to the master plate and then spun at 500g for 5 minutes at 4°c. Pelleted debris from fibrin was lifted from the bottom, and the plates were spun again at 1200 rpm for 10 minutes. Assays were performed in duplicate for validation unless in cases where volumes were low, and the average value was generated. The lower limit of detection (LLD) was 5 pg/ml for IL2 and 15 pg/ml for TNF.

### Variables and Definitions

#### Cough Aerosol Culture Status:

Cough aerosol culture-positive status (CAC +) was defined by culture growth on any CASS plates. Participants were considered cough aerosol culture-negative (CAC−) if no CASS plates were positive for *Mtb* growth and no more than two of the six plates were discarded as contaminated with fungal or bacterial overgrowth.

#### IGRA measures andpositivity:

*IGRA* positivity was determined using standard QFT-Plus interpretations from the manufacturer with responses dichotomized as positive (>0.35 IU/mL, and ≥25% of Nil value) or negative (<0.35 IU/mL, or≥0.35 IU/mL and <25% of Nil value, with a response to the mitogen control ≥0.5 IU/mL) after excluding indeterminate responses. [34]

#### Defining positivity by TNF and IL-2 responses.

We simulated the IGRA criteria to define positivity by TNF and IL-2 responses. TNF and IL-2 responses were considered positive if all of the following criteria were met: (1) TB1 ≥ LLD (5 pg/ml for IL2 and 15 pg/ml for TNF); (2) TB1-Nil ≥ LLD; (3) TB1-Nil ≥ 25% of sample Nil. TNF and IL-2 responses were considered negative if the patient did not meet the abovementioned criterion with a response to the mitogen control that was ≥ LLD.

#### Mtb infection Endpoints.

*We* categorized participants as having *Mtb* infection (latent TB infection, LTBI) (inferred based on evidence of *Mtb*-specific CD4 T-cell responses) in four ways based on the above criteria of defining positivity: IFN-γ status based on the standard IGRA criteria, IL2 status, TNF status, and finally, using a composite endpoint of any ESAT6/CFP10 response among IFN-γ, IL2, and TNF, “composite measure.” Using the IGRA criteria, we excluded 6 HHC with indeterminate and 4 HHC missing QFT-Plus test results in categorizing participants as having *Mtb* infection. However, these participants were assessed for IL2 and TNF responses.

### Statistical analysis

#### Index and Household Contact Characteristics Analyses:

We compared baseline characteristics of HHC by IGRA status using Pearson’s chi-square or Fisher’s exact tests and the t-test or Wilcoxon rank-sum as applicable. We used bivariate logistic regression with random intercepts, without adjustment, to account for clustering for index participant characteristics.

#### IFN-γ TNF and IL-2 responses in HHC:

IFN-γ, TNF, and IL-2 concentrations in the TB1 and mitogen tubes were background-corrected before analysis by subtracting the concentration from the negative control (nil) sample. Correlation plots were created for the TB1 minus nil (TB1-Nil) responses to assess potential associations between cytokines (**Supplementary Figure**).

#### Associations with cytokine responses and positivity in household contacts:

Our primary outcome of interest was cytokine responses and positivity in household contacts, and our primary predictor was the cough aerosol culture status of the index participant. Using the defined endpoints, cytokine responses were dichotomized as positive or negative (excluding indeterminate and unclassified responses). Wilcoxon rank sum test with continuity correction was used to assess the association of cytokine concentrations (pg/ml) with cough aerosol culture status, cavitary disease, and other categorical variables. Spearman’s correlation was used to examine the association of cytokine responses with continuous and ordinal variables. We further assessed associations between predictors and outcomes using bivariate and multivariable logistic regression models with random intercepts to account for clustering by index participants. Multivariable models included covariates with statistically significant results based on p-values <0.10 in bivariate analyses after assessing multicollinearity between our independent variables using variance inflation factors. We removed any explanatory variables with evidence of multicollinearity from the model. We introduced an interaction term between cavitary disease and CAC status to test the difference between these associations by the presence of cavitary disease. Significance was set at a α = 0.05. Missing data were addressed by pairwise deletion to reduce the chance of biased estimates. Analyses were performed using STATA V.17 (StataCorp, College Station, TX) and RStudio: Integrated Development Environment for R version 4.4.0 (2024-04-24).

## Results

### Participant Characteristics and Mtb-speci fic TNF and IL-2 Responses in HHC

Among 230 HHCs, 58.7% (n=135) were female, the median age was 14.9 years (interquartile range [IQR], 6.5–32.0), the median BMI was 20 kg/m2 (IQR 16.6–25.4), and the median MUAC was 23 cm (IQR 16.5–27.5). Only 3.5% (n=8) were PLWH, 9.0% (n=19) reported alcohol use, and 3.3% reported tobacco use. *Mtb* infection (IGRA positive) was detected in 58.6% of participants (129/220, excluding 6 with indeterminate and 4 with missing results). Sixty-five percent (65%) of households had two or more HHCs for each index participant (Table 1), and 29 of 92 (13%) had both IGRA positive and negative HHCs. Compared to HHCs with a negative IGRA, HHCs with a positive IGRA were older (median 17.4 vs. 12.8 years, *p*=0.02) and had larger MUAC (median 23.0 vs. 22.0 cm, p=0.03). We observed no difference in IGRA positivity rates as the number of contacts increased in households. (**Supplementary Table 1**). Characteristics of the index participants have been described previously. [33]

**Figure 1:**
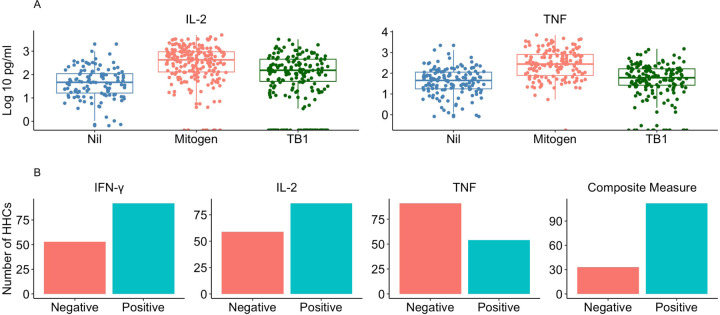
Household contact IL2 and TNF cytokines measured from the QFT-Plus supernatants. **A:** IL2 and TNF concentrations (pg/mL) measured from the QFT-Plus supernatant of the nil, mitogen, and TB1 (ESAT6/CFP10 stimulated) tubes. We observed no precise bimodal distribution for ESAT6/CFP10 IL-2 and TNF responses **B**: Positivity results by cytokine. 129 of 220 (58.6%) HHC were positive by QFT-Plus IFN-γ interpretation, 121 of 210 (57.6%) HHC were regarded positive by IL-2, and 55/151(36.4%) HHC were regarded positive by TNF. Using a combined endpoint of any response among IFN-γ+IL-2+TNF+, we identified a total of 154 of 227 (67.8%) with an antigen-specific response, a 9.2% increase from using QFT-Plus IFN-γ interpretation alone.

We analyzed levels of TNF and IL-2 in QFT-Plus supernatants among HHCs of TB index cases. TNF responses demonstrated moderate background (median 34.4 pg/mL, IQR 12.5–99.5), strong mitogen responses (mitogen-nil 187.5 pg/mL, IQR 38.4–709.2), with a small margin between TB1 and nil levels (median 3.9 pg/mL), and no precise bimodal distribution (TB1-nil range 0–845.3, IQR 0–45.9), with 57% participants having TB1-nil >0 (Figure 1A **and Supplementary Table 2**), and 47% (71/151) having TB1-nil > 25% of the Nil. IL2 responses had low background (median nil 6.2 pg/mL, IQR 0–50.5), strong mitogen responses (mitogen-nil 305.1 pg/mL, IQR 59.2–851.9), with a wide margin between TB1 and nil levels (median 35.4 pg/mL), and no precise bimodal distribution for TB1-nil (TB1-nil range 0–2795.6 pg/mL, IQR 0–195.0) with 63.8% participants having TB1-nil > 0 (Figure 1A **and Supplementary Table 2**), and 59% (124/210) participants having TB1-nil > 25% of the Nil. For the TB1-Nil responses, we found a weak correlation between IL-2 and TNF (r =0.165, *p*=0.04) and between QFT-Plus IFN-γ and TNF (r =0.179, *p*= 0.03). QFT-Plus IFN-γ and IL-2 had moderate correlation (r =0.459, *p*<0.001). (**Supplementary Figure**). In a secondary analysis, we found that IL-2 concentrations (TB1-nil) were significantly higher among IGRA positive HHC compared to IGRA negative (150.6 pg/mL IQR 64.33–393.8 vs 0 pg/mL IQR 0–0; *p*<0.0001) (**Supplementary Table 3**). In contrast, TNF concentrations (TB1-nil) were not different (4.47 pg/mL IQR 0–33.09 vs 2.25 pg/mL IQR 0–64.5, *p*=0.52) (**Supplementary Table 3**). Together, these data show that IL-2 and TNF responses among HHC of TB participants lack a bimodal distribution and are not highly correlated with each other or with QFT-Plus IFN-γ.

### Positivity by Mtb-specific TNF and IL-2 responses in HHC

Modeling the positive/negative criteria of QFT-Plus, we defined positivity thresholds for TNF and IL-2 responses in HHC. The median concentrations (IQR) among participants regarded positive by IL-2 and TNF were 152.0 (69.5–412.8) and 71.1 (38.0, 153.3), respectively. Compared with 58.6% (129 of 220) HHC considered positive by QFT-Plus interpretation, 57.6% (121 of 210) HHC were regarded positive by IL-2 (eight being negative and one having an indeterminate result by QFT-Plus IFN-γ) and 36.4% (55/151) HHC were considered positive by TNF (19 being negative by QFT-Plus IFN-γ) (Figure 1B). Eight (8) HHCs considered positive by QFT-Plus IFN-γ were regarded negative by both IL-2 and TNF, and 34 of 147 (23%) HHCs with complete data for the three cytokines were positive by all three criteria (IFN-γ+IL-2+TNF+). Using a combined endpoint of any response among IFN-γ, IL-2, and TNF, we identified 25 additional HHC with evidence of an ESAT6/CFP10-specific response (6 by IL-2 alone, 17 by TNF alone, and 2 by IL-2 and TNF). This brought the total classified as having *Mtb* infection to 154 of 227 (67.8%), a 9.2% increase from using QFT-Plus IFN-γ interpretation alone (Figure 1B).

We compared HHCs with a positive QFT-Plus result (IGRA+, N=129) with the 25 HHCs who had negative QFT-Plus (IGRA-) but had positive IL-2 or TNF (IGRA-&IL-2 +/TNF+). IGRA-&IL-2+/TNF+ HHCs were younger (median 11.5 IQR 5.3–23.4 vs. 17.4 IQR 9.1–35.3 years, *p*=0.04); other characteristics did not differ. Additionally, we compared HHCs that were positive by all three criteria (IFN-γ+IL-2+TNF) with HHCs that were positive by one or two criteria and found no differences in the characteristics (**Supplementary Table 4**). Together, these data suggest that IL-2 and TNF endpoints improve the detection of *Mtb* infection in HHC of TB participants compared with an IFN-γ endpoint alone.

### Association of index case CAC Status with *Mtb*-specific IFN-γ, TNF, and IL-2 responses in HHC

**Figure 2:**
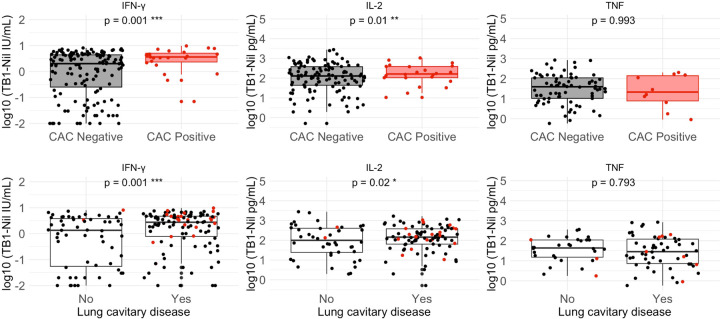
Distribution of household contact cytokine concentrations stratified by index case Cough Aerosol Culture (CAC) and chest x-ray lung cavitation statuses. Scatter points represent individual household contact cytokine measures, and the colors represent the index case CAC status (black, CAC negative, and red, CAC positive). **A:** Household contact cytokine concentrations stratified by index case CAC status. IL-2 and QFT-Plus IFN-γ concentrations were significantly higher among HHC of CAC-positive than those of CAC-negative index participants. **B:** Household contact cytokine concentrations stratified by index case chest x-ray lung cavitation status. IL-2 and QFT-Plus IFN-γ concentrations were significantly higher among the HHC of index participants with, compared to those without, lung cavitary disease.

Next, we examined the impact of index case aerosolized *Mtb* on HHC TNF and IL-2 compared with QFT-Plus IFN-γ responses. Median IL-2 concentrations (TB1-nil) were significantly higher among HHC of CAC+ compared to those of CAC− index participants (142.06 pg/mL IQR 32.58–381.88 vs. 21.21 pg/mL IQR 0–158.56; p=0.01). The same trend was observed with QFT-Plus IFN-γ concentrations (TB1-nil) comparing HHC of CAC+ and CAC− index participants (3.42 IU/mL IQR 0.81–4.68 vs 0.49 IU/mL IQR 0–3.50; p=0.001) (Table 2, Figure 2A). In contrast, TNF concentrations were not different (3.96 pg/mL IQR 0–45.87 vs 1.76 pg/mL IQR 0–28.53, p=0.99) (Table 2). We also assessed the association of cavitary lung disease and other measures of bacillary burden with IFN-γ, IL-2, and TNF responses in HHCs. IL-2 (*p*=0.02) and IFN-γ (*p*=0.001), but not TNF (p=0.79), were positively associated with cavitary lung disease regardless of CAC status (Table 3, Figure 2B). We observed no association between TNF, IL-2, and IFN-γ concentrations and bacillary burden measured by GeneXpert cycle threshold (*p*-values 0.50, 0.79, and 0.11), Xpert Grade (*p*-values 0.06, 0.40, and 0.15), sputum smear grade (*p*-values 0.05, 0.19, and 0.05) and TTD of *Mtb* (*p*-values 0.66, 0.44, and 0.05), respectively.

We then examined whether aerosolized *Mtb* was associated with *Mtb* infection, as defined by various positivity endpoints using ESAT6/CFP10 responses for TNF, IL-2, and QFT-Plus IFN-γ (IGRA). We used mixed-effects logistic regression models to account for clustering by index participants. Comparing HHC of CAC+ and CAC− index participants, exposure to a CAC+ index was associated with *Mtb* infection when using IL2 (84.6% vs 53.8%, odds ratio [OR] 6.98, 95 % confidence interval [CI] 1.33–36.75, *p*=0.02) and IGRA (88.0% vs 54.9%, OR 11.5, CI 1.68–78.7, *p*=0.01), but not TNF (35.3% vs 36.6%, OR 0.96, CI 0.30–3.09, *p*=0.94) endpoints independently (Table 4). Using any positive ESAT6/CFP10-specific cytokine response (composite criteria including IL-2, TNF or IFN-γ), exposure to a CAC+ index was still associated with *Mtb* infection (88.5% vs 66.2% comparing CAC+ and CAC−, respectively, OR 5.37, CI 1.07–27.0, *p*=0.04). This also held when the endpoint was narrowed to include any positive of QFT-Plus or either of the two non-IFN-γ endpoints: using IL2/QFT-Plus (OR 5.70, CI 1.10–29.56, *p*=0.04) and TNF/QFT-Plus (OR 6.48, CI 1.27–33.11, *p*=0.03) (Table 4). In multivariable models, the observed associations with CAC status were maintained, and we observed that chest X-ray cavitation modified the effect of CAC-positive status on all *Mtb* infection endpoints (Table 4). Together, these data suggest that IFN-γ and IL-2 endpoints are associated with aerosolized *Mtb* independently or as part of a measure comprising any positive ESAT6/CFP10-specific cytokine response, including IL-2, TNF, or IFN-γ.

### Association of index case HIV co-infection with *Mtb-* specific IFN-γ, TNF, and IL-2 responses in HHC

We also examined the impact of index case HIV co-infection on *Mtb* infection as defined by various positivity endpoints using ESAT6/CFP10 responses for TNF, IL-2, and QFT-Plus IFN-γ. *Mtb* infection status differed by index case HIV co-infection status for the QFT-Plus IFN-γ endpoint (35.7% of PLWH vs 62.3% of persons without HIV with *Mtb* infection by a positive QFT-Plus, OR 0.17, CI 0.04–0.82, *p*=0.03), but not TNF or IL-2 endpoints (Table 5) Together, this data suggests that HIV co-infection may be associated with significantly lower odds of *Mtb* infection in HHC when the QFT-Plus IFN-γ endpoint is used alone.

## Discussion

We assessed the utility of *Mtb*-specific TNF and IL-2 (from supernatants of the QFT Plus TB1 antigen tube) in improving *Mtb* infection detection in HHCs of TB participants and the effect of index case aerosolized *Mtb* and HIV co-infection on cytokine responses. We had several primary findings, including that 9.2% of HHCs had an antigen-specific TNF and IL-2 response, yet no response by QFT-Plus IFN-γ; aerosolized *Mtb* exposure was associated with IL-2 and IFN-γ, but not TNF, in HHCs; and HIV co-infection in the index was associated with IFN-γ responses, but not IL-2 or TNF responses in HHCs.

Our findings of weak to moderate correlation of the three cytokines suggest that each may have complementary roles in *Mtb* infection detection. Like QFT-Plus IFN-γ, ESAT6/CFP10 responses for IL-2 and TNF lacked a bimodal distribution, which can be used to define positivity thresholds. Modeling the positive/negative criteria of QFT-Plus, [34] we identified 9.2% of HHCs that were Mtb uninfected by traditional criteria (lacked evidence of sensitization to *Mtb* antigens based on in vitro release of IFN-γ) yet had evidence of sensitization to *Mtb*-specific TNF and IL-2. We did not test the criteria for defining positivity thresholds in individuals from a non/low-endemic area. However, we observed that for IL-2 concentration, the lower quartile among participants inferred to have *Mtb* infection (69.5 pg/mL) approximated the threshold (66 pg/mL) identified for discriminating TB-infected from uninfected persons in a low-endemic area in a previous study. [9] Our findings extend past evidence where we and others highlight that IFN-γ independent T cell responses may identify *Mtb* exposure not reflected by the current IFN-γ based diagnostic tests. [10–12, 21, 22]

We also found that the additional HHCs identified were more likely to be younger. Recently, we found that Mtb-specific non-IFN-γ cytokine responses have improved sensitivity in detecting *Mtb* infection in HIV-exposed infants compared with IGRA. [22] Our new data in *Mtb*-exposed HHCs suggest that IL-2 and TNF may have a role in detecting *Mtb* exposure in younger persons, such as children, who have reduced sensitivity to IGRA. [35, 36] Our findings and past evidence have implications for developing innovative tests with potential use in TB diagnostics in line with The End TB Strategy. [37]

The role of IFN-γ, IL-2, and TNF in TB pathogenesis has been studied extensively. Distinct profiles of *Mtb-*specific IFN-γ, IL-2, and TNF CD4+ T-cells are thought to be associated with different *Mtb* infection states and the related bacterial loads within the host. [38–40] *Mtb*-specific IL-2 and TNF have also been detected in individuals heavily exposed to *Mtb* without evidence of an immunologic response based on traditional in vitro release of IFN-γ. [10, 11, 21] CAC is an important measure of TB infectiousness, [26–28] given it is a direct measure of *Mtb* aerosolization supported by existing evidence of the crucial role of aerosols in infectivity and immunopathology. [23, 41, 42] Data on the differential cytokine expression by levels of exposure is sparse and limited to the IFN-γ response. [26, 27, 43] In keeping with this past evidence, we recently found that the CAC status of the index predicted QFT Plus positivity in HHCs. [33] Our new data extends these findings to show that IL-2 and IFN-γ, but not TNF, independently or as part of a measure comprising any positive ESAT6/CFP10-specific cytokine response, are associated with aerosolized *Mtb* (CAC status).

Consistent with previous findings suggesting a lower likelihood of *Mtb* transmission from PLWH compared to persons without HIV, [29–31, 44] we recently found that HIV coinfection in the index predicted a negative QFT Plus in HHCs. [33] In the current study, we extend these findings to assess the association between HIV coinfection in the index and HHC *Mtb* infection defined by various positivity endpoints using ESAT6/CFP10 responses for TNF, IL-2, and QFT-Plus IFN-γ. As reported previously, [33] there was a negative association between HIV co-infection in the index and *Mtb* infection in HHC using the QFT-Plus IFN-γ endpoint independently. Although PLWH had lower odds of having HHCs with *Mtb* infection using the IL-2 endpoint or the composite measure comprising any positive ESAT6/CFP10-specific cytokine response, there was no evidence of a significant association. There have been contradictory reports in the past, with other studies finding no evidence for an association between HIV coinfection and *Mtb* transmission. [30, 32] Together, these data suggest that additional studies using different methodologies, including exploring the role of non-IFN-γ responses in confirming *Mtb* infection and using Mtb-specific antigens beyond ESAT-6/CFP10, may provide further insights into *Mtb* transmission dynamics among PLWH.

Our study has several limitations. TNF and IL-2 lacked a bimodal distribution, limiting the ability to define appropriate cutoffs. As this was like QFT-Plus IFN-γ, we mimicked the QFT-Plus criteria in defining positivity. Given that the IGRAs cutoff is also suggested to have an uncertainty zone and that there is a high likelihood of conversion within a year of exposure, [45–47] these cutoffs may need optimization in future research. We had a small number of index participants who were CAC positive and living with HIV, limiting the ability to study associations comprehensively. In this analysis, we do not report follow-up data to show evidence of protection from TB or risk of TB progression based on the various ESAT6/CFP10-specific cytokine responses and CAC status. We had some missing data points, which we addressed by pairwise deletion to reduce the chance of biased estimates. Despite these limitations, our study has strengths, including using multiple diagnostic approaches to maximize test sensitivity in diagnosing possible *Mtb* infection in HHCs and determining, for the first time to our knowledge, associations with *Mtb* *ae*rosolization and HIV coinfection.

In summary, we show that IL-2 and TNF, in addition to QFT outcomes, may provide additional benefits in *Mtb* infection diagnosis and phenotyping, help predict *Mtb* transmission events, and provide further insights into *Mtb* transmission dynamics among PLWH. Our findings have implications for developing innovative tests with potential use in TB diagnostics in line with The End TB Strategy for moving forward to the 2035 targets. We recommend further studies to optimize the diagnosis of *Mtb* infection using non-IFN-γ cytokines and study associations with CAC status and HIV coinfection.

## Figures and Tables

**Table 1:** Characteristics of Household Contacts and Index Participants

Household contact (HHC) characteristics	Total (N=230)
Female gender (n, %)	135 (58.7%)
HHC Age, years (Median, IQR)	14.9 (6.5, 32.0)
BMI, kg/m^2^ (median (IQR)^†^	20 (16.6, 25.4)
MUAC, cm (median, IQR)^††^	23 (16.5, 27.5)
PLWH^†††^	8 (3.5%)
Alcohol use (N=212)	19 (9.0%)
Smoking status (N=212)	7 (3.3%)
Shared bedroom with index (N=215)	144 (67.0%)
Previously treated for TB (N=216)	11 (5.1%)
Positive QuantiFERON (QFT) Result in HHC (N=220) *	129 (58.6%)
Index TB participants with whom HHC Resided	Total (N=92)
Female gender (n, %)	35 (41.2%)
Index Age, years (Median, IQR) (N=91)	31.1 (25.4, 41.5)
BMI (N=90)	19.5 (18.0, 22.2)
MUAC, cm (N=90)	23.5 (21.7, 25.7)
PLWH (N=91)	13 (14.3%)
Lowest Ct value (Median, IQR) (N=82)	19.5 (16.7, 24.5)
Xpert Grade (n, %) (N=91)	
Grade 1 (Trace)	10 (11.0%)
Grade 2 (Very Low)	7 (7.7)
Grade 3 (Low)	19 (20.9)
Grade 4 (Medium)	32 (35.2)
Grade 5 (High)	23 (25.3)
Smear grade (n, %) (N=91)	
Negative	18 (19.8%)
Scanty	10 (11%)
(+) 1	25 (27.5%)
(+) 2	19 (20.9%)
(+) 3	19 (20.9%)
CAC +^††††^	11 (12%)
TTD, days (Median, IQR) (N=82)^†††††^	6 (4, 11)
CXR Cavitary disease (N=91)	55 (60.4%)
CRP (Median, IQR) (N=88)	55.1 (14.9, 86.9)
Number of contacts for each index participant (% of households)	
1 HHC	32 (34.8%)
2 HHC	19 (20.7%)
3 HHC	16 (17.4%)
4 HHC	16 (17.4%)
5 HHC	6 (6.5%)
6 HHC	3 (3.3%)
Index participants with at least one HHC having positive QFT Result (n, %)	62 (67.4%)

* 6 HHC had Indeterminate QFT-Plus test results and 4 HHC had missing QFT-Plus test results. QFT positivity is based on the remaining 220;

† BMI, Body mass index, IQR, interquartile range;

†† MUAC, Mid-Upper Arm Circumference;

††† PLWH, Person Living with HIV;

†††† CAC+, Cough Aerosol Culture positive/*Mtb* growth;

††††† TTD, Time to detection of *Mtb* growth in liquid media; Not all participants had complete data, the total with complete data is indicated where it differs from 230 in HHC or 92 in index participants.

**Table 2:** Cytokine levels in household contacts by index case CAC status

	N^a^	Index CAC − (Median, IQR) +	N^b^	Index CAC+ (Median, IQR)^†^	p-value*
**Baseline (unstimulated) cytokine levels**					
IFN-γ (IU/mL)	200	0.19 (0.05, 0.59)	26	0.21 (0.13, 0.44)	0.323
TNF (pg/mL)	141	38.74 (13.2, 107.0)	18	26.42 (6.91, 63.19)	0.401
IL-2 (pg/mL)	191	4.40 (0, 48.70)	26	19.86 (0, 67.56)	0.300
**Cytokine levels in response to mitogen**					
IFN-γ (IU/mL) **	200	5.95 (3.81, 7.19)	26	6.64 (4.6, 8.63)	0.126
TNF (pg/mL) **	121	186.12 (37.70, 759.35)	17	198.84 (61.59, 582.65)	0.667
IL-2 (pg/mL) **	178	299.93 (56.44, 839.2)	26	403.12 (118.31, 864.52)	0.529
**Cytokine levels in response to TB1 antigens**					
IFN-γ **(N=226)	200	0.49 (0, 3.50)	26	3.42 (0.81, 4.68)	**0.001**
IL-2**(N=210)	184	21.21 (0, 158.56)	26	142.06 (32.58, 381.88)	**0.010**
TNF**(N=151)	134	3.96 (0, 45.87)	17	1.76 (0, 28.53)	0.993

N includes only HHC with the various cytokine measures, HHCs with indeterminate IGRA results are included;

a Total HHCs exposed to index with negative cough aerosol cultures (CAC);

b Total HHC exposed to index with positive CAC;

† Cytokine levels in HHC exposed to index participants with negative and positive CAC;

* Wilcoxon-rank sum,

** background corrected TB1-nil (IL-2 and TNF pg/mL, IFN-γ IU/mL).

**Table 3:** Cytokine levels in response to TB1 antigens in household contacts by index case cavitation status

	Cavitation - (N=89) (Median, IQR)^†^	Cavitation + (N=136) (Median, IQR)^†^	p-value*
IFN-γ** (N=225)	0.05 (0, 2.83)	1.805 (0.02, 4.38)	**0.001**
IL-2** (N=209)	4.42 (0, 140.2)	78.16 (0, 200.37)	**0.020**
TNF** (N=150)	4.47 (−24.47, 45.91)	2.80 (−3.16, 44.28)	0.793

† Cytokine levels in HHC exposed to index participants without cavitation on chest x-ray (cavitation−) and with cavitation (cavitation+);

* Wilcoxon-rank sum,

** background corrected TB1-nil (IL-2 and TNF pg/mL, IFN-γ IU/mL)

**Table 4:** Effect of index case CAC status on *Mtb* infection in household contacts

Mtb infection Endpoint	CAC− Exposure		CAC+ Exposure		p-value*	OR (95% CI) *	aOR (95% CI) *
	N^a^	(n, %)^b^	N^c^	(n, %)^d^			
**Any ESAT6/CFP10 response**							
QFT-Plus (IFN-γ)	195	107 (54.9%)	25	22 (88.0%)	**0.013**	**11.50 (1.68, 78.7)**	**29.35 (1.83, 471.40)** ^†^
IL-2	184	99 (53.8%)	26	22 (84.6%)	**0.022**	**6.98 (1.33, 36.75)**	**12.64 (1.87, 85.40)** ^††^
TNF	134	49 (36.6%)	17	6 (35.3%)	0.944	0.96 (0.30, 3.09)	-
**Composite measure** **	201	133 (66.2%)	26	23 (88.5%)	**0.041**	**5.37 (1.07, 27.0)**	**11.2 (1.55, 80.21)** ^††††^
**Any 2 ESAT6/CFP10 response including QFT-Plus (IFN-γ)**							
QFT-Plus (IFN-γ) orIL-2	201	116 (57.7%)	26	22 (84.6%)	**0.038**	**5.70 (1.10, 29.56)**	**14.91 (1.63, 136.28)** ^†††^
QFT-Plus (IFN-γ) or TNF	200	126 (63.0%)	26	23 (88.5%)	**0.025**	**6.48 (1.27, 33.11)**	**13.6 (1.89, 98.63)** ^††††^

CAC, Cough Aerosol Culture; N includes only HHC with the various cytokine measures;

a Total HHC exposed to index with Negative CAC;

b Number and percentage of HHC with a positive ESAT6/CFP10 response as specified in the respective row among those exposed to CAC− index;

c Total HHC exposed to index with Positive CAC;

b Number and percentage of HHC with a positive ESAT6/CFP10 response as specified in the respective row among those exposed to CAC+ index;

* Mixed-effects logistic regression;

** (QFT-Plus IFN-γ+ IL-2+TNF+);

† adjusted for HHC age, index HIV status, index cavitary disease, index CRP;

†† adjusted for index cavitary disease;

††† adjusted for HHC age and index cavitary disease;

†††† adjusted for index cavitary disease

**Table 5:** Effect of index case HIV status on *Mtb* infection in household contacts

Mtb infection Endpoint	Exposure to HIV− index		Exposure to HIV+ index		p-value*	OR (95% CI) *
	N^a^	(n, %)^b^	N^c^	(n, %)^d^		
**Any ESAT6/CFP10 response**						
QFT-Plus (IFN-γ)	191	119 (62.3%)	28	10 (35.7%)	**0.027**	**0.17 (0.04, 0.82)**
IL-2	179	107 (59.8%)	30	14 (46.7%)	0.262	0.45 (0.11, 1.81)
TNF	131	48 (36.6%)	19	7 (36.8%)	0.934	1.05 (0.34, 3.22)
**Composite measure** **	196	139 (70.9)	30	17 (56.7%)	0.216	0.49 (0.15, 1.53)
**Any 2 ESAT6/CFP10 response including QFT-Plus (IFN-γ)**						
QFT-Plus (IFN-γ) + IL-2	196	123 (62.8%)	30	15 (50.0%)	0.241	0.44 (0.11, 1.73)
QFT-Plus (IFN-γ) + TNF	196	135 (68.9%	29	14 (48.3%)	0.086	0.36 (0.11, 1.15)

N includes only HHC with the various cytokine measures;

a Total HHC exposed to index with Negative HIV status;

b Number and percentage of HHC with a positive ESAT6/CFP10 response as specified in the respective rows among those exposed to HIV- index;

c Total HHC exposed to index with Positive HIV status;

b Number and percentage of HHC with a positive ESAT6/CFP10 response as specified in the respective rows among those exposed to index with Positive HIV status;

* Mixed-effects logistic regression;

** (QFT-Plus IFN-γ+ IL-2+TNF+)

## Data Availability

The data supporting this study’s findings are not publicly available due to privacy policy but will be available from the corresponding author upon reasonable request.
